# Book Reviews

**Published:** 1895-09

**Authors:** 


### *BOOK REVIEWS.*

---

MEDICAL GYNECOLOGY. A Treatise on the Diseases of Women from the Standpoint of the Physician. By Alex. J. C. Skene, M.D., etc., New York. D. Appleton & Co., New York.

The author of this book has strong claims upon the gratitude of the profession, for original work in various directions. His contributions to the literature of gynecology will transmit his name to an admiring posterity. But, for practical usefulness, no work which he has written equals the volume before us.

The surgical tendency of gynecology has been so strongly marked of late years, that there is danger of losing sight of the fact that there is a medical side to this branch of practice. The idea of curing pelvic diseases in women by hygienic and therapeutic measures would appear to be almost an obsolete one, to an observer of current medical literature. While it is true that conservative gynecologists treat the larger proportion of their cases without recourse to surgery, it is equally true that it is rare for any but surgical cases to find a place in the medical journals and society transactions. The surgical tendency exists, therefore, more in appearance than in reality.

The credit which is due to Dr. Skene for writing this book lies not so much in any new or original methods of treatment, or new views of pathology or causation, as in showing to the profession at large the extensive resources upon which specialists are in the habit of relying for the relief and cure of a large proportion of their cases, and thus correcting the prevailing impression that gynecology is simply a branch of surgery. This book will be a surprise to two classes of medical men. First, to those who decry specialism and deprecate the so-called operative tendency of specialists in this branch; second, to those who, not choosing to do any surgery themselves, send all their cases of diseases of women to the specialist, with the expectation that he will operate upon them, and thereby effect an immediate cure. The two extremes of skepticism and credulity are equally remote from the truth.

This work is not a mere manual of treatment. It is a philosophic treatise upon woman in relation to all phases of her physical, mental, and moral environment, from puberty to old age. *Felix qui potuit rerum cognoscere causas.* The author has attained to a degree of felicity in this respect which is seldom equaled in works upon this subject. To preventive treatment is given a greater prominence than to curative treatment, and thus the general practitioner, who "hates gynecology" and is only too glad to refer his cases to the convenient specialists, will find in this book a means of avoiding this objectionable class of practice without sacrificing his *clientele.*

Without going into detail, it may be truly said, that every chapter is replete with wise suggestions, which will set the observant man to thinking, and which will give a new aspect to female diseases. In this way the book will advance the cause of conservatism in gynecology on the one hand, and on the other will lead to a proper appreciation of the true value of this specialty. V. O. H.

---

TWENTIETH CENTURY PRACTICE. An International Encyclopedia of Modern Medical Science. By Leading Authorities of Europe and America. Edited by Thomas L. Stedman, M.D., New York City. In Twenty Volumes. Volume III. Occupation Diseases, Drug Habits, and Poisons. New York: William Wood & Company. 1895.

The third volume of this great work discusses in the usually thorough manner a variety of subjects. Alcoholism and Drug Habits are described at great length by one of the best authorities on these subjects, Dr. Norman Kerr, of London, author of the well known work on "Narcomania." Dr. George F. Shrady, the talented editor of the *Medical Record*, gives, in a very good article, the phenomena and treatment of shock. The articles on Seasickness, Heat-stroke, and Frost-bite are well written by Dr. Albert H. Gihon, of the United States Navy, whose long experience on the sea especially qualifies him to speak on these subjects. We have never seen "Seasickness" so well presented. Professor Small, of Ottawa, and Professor Stewart, of Montreal, deal with the subject of Toxicology, describing the effects of the most common and important poisons, and the methods of treatment, with which the practitioner should be familiar. The most elaborate article in the volume, and at the same time practical, is that on Diseases of Occupation, by Dr. James Hendrie Lloyd, of Philadelphia. The author goes very fully into the specific diseases of workers in lead, arsenic, mercury, phosphorus, zinc, carbon, iodine, illuminating gas, tobacco, etc., the effects of injurious environment, such as rarified and compressed air, heat and cold, bad sanitation and ventilation, occupation neuroses, such as writer's and telegrapher's cramp, neurasthenia from overwork, the fatigue neuroses, etc.; a very complete and exhaustive article of 185 pages. Professor Councilman, of Harvard, has a good article on Osteomalacia, and Dr. von Liebig, of Munich, an interesting one on Mountain Sickness.

Every physician will do well to add these volumes to his library. They will be a whole storehouse of knowledge, supplied by our best authorities.

---

SYSTEM OF SURGERY. Edited by Frederic S. Dennis, M.D., Professor of the Principles and Practice of Surgery, Bellevue Hospital Medical College, etc.; assisted by John S. Billings, M.D., Deputy Surgeon-General U. S. A. Volume II. Published by Lea Brothers & Co., 706–710 Sansom street, Philadelphia.

This volume, the second of the series, comprises able and complete descriptions of Minor, Plastic, and Military Surgery, Diseases of the Bones, Orthopedic Surgery, Aneurism, Surgery of the Arteries, Veins, and Lymphatics, Diseases and Injuries of the Head, Surgery of the Spine, and Surgery of the Nerves. The list of authors includes some of the best-known American teachers and surgeons, such as the author, Dr. Dennis, Dr. George R. Fowler, Dr. Virgil P. Gibney, Dr. W. W. Keen, Dr. Nicholas Senn, Dr. Roswell Park, Dr. L. A. Stimson, and others. The most important sections of the volume are those on Diseases of the Bones, by Dr. Senn; Orthopedic Surgery, by Dr. Gibney; Surgery of the Spine, by Dr. Keen; and Diseases and Injuries of the Head, by Dr. Park. The latter is hardly less than a treatise, comprising nearly three hundred pages, and is very exhaustive. Dr. Keen's article on The Spine is also deserving of special commendation. The article by Dr. Wharton on Minor Surgery is almost a reproduction of the popular text-book by the same author. The article on Military Surgery and the Care of the Wounded on the Battle-field, by Dr. Forwood, Deputy Surgeon-General, U. S. A., will be of great interest and value to army surgeons.

This work is proving to be an exceedingly valuable contribution to our surgical literature, and highly creditable to both authors and publishers.

---

The Pocket Materia Medica and Therapeutics. A Résumé of the Action and Doses of all Officinal and Non-Officinal Drugs now in common use. By C. Henri Leonard, A.M., M.D., Professor of the Medical and Surgical Diseases of Women and Clinical Gynecology in the Detroit College of Medicine. Second Edition. Price, post-paid, $1.00. The Illustrated Medical Journal Co., Publishers, Detroit, Mich.

The second edition of this popular therapeutic work has had sixty-seven pages added to it, besides typographical errors corrected, etc. A new and complete cross-index has been prepared, which renders the quick finding of a non-familiar drug possible.

The descriptive arrangement of the drugs is as follows: Alphabetically the drug, with its pronunciation (officinal or non-officinal standing indicated), genitive case-ending, common name, dose, and metric dose. If a plant, the part used, habitat, natural order, botanic description, with alkaloids if any; if a mineral, its chemical symbol, atomic weight, looks, taste, how found, its peculiarities. Then the action and uses of the drug or compound, its antagonists, its incompatibles, its synergists, and then antidotes. Then follow its officinal and non-officinal preparations, with their medium and maximum doses. Altogether it is a handy volume for physician, druggist, or student, and will be frequently appealed to if in one's possession.

---

Exercise and Food for Pulmonary Invalids. By Charles Denison, M.D., Denver, Colorado; Professor of Diseases of the Chest and Climatology, University of Denver, etc. The Chain & Hardy Company, Publishers, Denver. Price 35 cents.

This little book seeks to describe those forms of light exercise which may be used with advantage by those with weak chests and defective lung power; also to suggest a suitable dietary for the victims of lung diseases, particularly tuberculosis. The book is well written in a clear and simple style, and may be read with advantage by both physicians and patients.

